# Dysfunction of the Autophagy System and MDM2–p53 Axis Leads to the Accumulation of Amyloidogenic Proteins in Angelman Syndrome Models

**DOI:** 10.3390/ijms262211032

**Published:** 2025-11-14

**Authors:** Jacqueline Fátima Martins de Almeida, Martina Contestabile, Ilaria Tonazzini, Chiara De Cesari, Laura Baroncelli, Claudia Martini, Simona Daniele

**Affiliations:** 1 Department of Pharmacy, University of Pisa, 56126 Pisa, Italy; jacqueline.moraes@farm.unipi.it (J.F.M.d.A.); m.contestabile@student.unisi.it (M.C.); claudia.martini@unipi.it (C.M.); 2 Istituto Nanoscienze, Consiglio Nazionale delle Ricerche (CNR)@NESTe, Piazza San Silvestro 12, 56127 Pisa, Italy; ilaria.tonazzini@cnr.it (I.T.); chiara.decesari@nano.cnr.it (C.D.C.); 3 Istituto Neuroscienze, CNR, Via Giuseppe Moruzzi 1, 56124 Pisa, Italy; laura.baroncelli@in.cnr.it

**Keywords:** Angelman syndrome, autophagy, protein accumulation, ubiquitin–proteasome system, amyloid proteins

## Abstract

Angelman Syndrome (AS) is a neurodevelopmental disorder caused by the deficiency of the UBE3A gene that for a E3 ligase protein part of the ubiquitin–proteasome system (UPS). Autophagy and UPS systems remove abnormal proteins, but any dysfunction in these processes can affect neuronal development and wellbeing. Herein, the involvement of the UPS/autophagy system in the regulation of intracellular signaling pathways related to toxic protein accumulation was investigated in cellular/mice AS models, silenced for UB3A (UB3A^−^). The main findings are as follows: (i) autophagy was upregulated in UBE3A^−^ cells with respect to control cells; (ii) a dysregulation of the AKT/mTOR pathway, linked to autophagy/synaptic development, was evidenced in cellular/animal models of AS with respect to controls; (iii) the ubiquitin ligase MDM2 was downregulated, and the tumor suppressor p53, normally inhibited by MDM2, enhanced its expression and transcriptional activity in UB3A^−^ cells with respect to controls. Finally, UB3A^−^ cells presented a significant alteration in the levels of β-amyloids with respect to control cells, and a reduction of α-synuclein levels, typical of neurodevelopmental disorder. Nevertheless, UB3A^−^ cells do not show evident morphological abnormalities. Overall, these data suggest that AS models presented an altered signaling pathway related to autophagy/UPS systems, potentially leading to the accumulation of toxic proteins affecting synaptic development.

## 1. Introduction

Angelman Syndrome (AS) is a neurodevelopmental disorder characterized by a set of symptoms such as severe cognitive impairment, epilepsy, sleep disturbances, feeding problems, speech impairments, strabismus, and a particular behavioral phenotype [1]. This phenotype results from different genetic mechanisms involving the chromosomal region 15q11.2–q13, ultimately leading to the loss of function of the maternally inherited gene that encodes for ubiquitin-protein ligase E3A (UBE3A). Defects, such as du/triplications, within the maternally-derived AS critical region result in autistic symptomatology, suggesting that the UBE3A gene has a crucial role in brain neurodevelopment [1,2].

The UBE3A gene encodes a ~100 kDa protein that functions as an E3 ligase in the ubiquitin–proteasome system (UPS), and also as a transcriptional regulator, for example as transcriptional co-activator for steroid hormone receptors [3]. The UPS is a large group of proteins responsible for the intracellular degradation in eukaryotic cells [3,4]. Ubiquitin E3 ligases are an important component of UPS, functioning by ligating ubiquitin to its target proteins and thereby regulating their proper (spatially and temporally controlled) localization or degradation [5]. The ubiquitination process is essential for maintaining cellular proteostasis, by regulating proteasomal degradation, selective autophagy, cell signaling, endocytosis, receptor trafficking, DNA damage response, cell cycle control, and programmed cell death [3].

Autophagy maintains cell homeostasis in the nervous system via the degradation of misfolded proteins, elimination of damaged organelles, and regulation of apoptosis and inflammation. Several ubiquitin E3 ligases are involved in autophagy regulation [6]. Studies have demonstrated that the loss of UBE3A interferes with pathways involved in the autophagy regulation, such as AMPK-mTOR (mammalian target of rapamycin) and the tumor protein p53 [7]; moreover, between the several UBE3A targets discovered, there are targets related to autophagy, such as Huntingtin-associated protein 1 (HAP), and regulator complex subunit p18 [8]. Defective protein metabolism has been recently reported in the brain of AS mice [8], resulting in stalled autophagy and accumulation of proteins [7]. Perturbations in the mTOR pathway have been implicated in general in autism spectrum disorders (ASDs) and, in particular, UBE3A deficiency has been associated with increased active p53 levels in the cerebellum of AS mice, finally favoring the induction of autophagy [9].

p53 is a direct UBE3A substrate but needs E6 interaction, so only the E6–UBE3A–p53 complex results in ubiquitination and proteasomal degradation of p53 [10], while in the absence of E6, MDM2 is the primary E3 ligase for p53. MDM2 acts by inhibiting the transactivation domains of p53 and targeting p53 toward ubiquitin-mediated degradation [11]. Analyses from a variety of mouse strains demonstrate that inappropriately activating p53 during embryonic and early post-natal development triggers a host of developmental defects. In these mouse strains, p53 hyperactivation has been achieved through mutations in Trp53, the gene encoding p53, or by defects in MDM2 (murine double minute 2), the main negative regulator of p53. In addition, MDM2+/− mice, which have elevated p53 activity due to reduced expression levels of MDM2, die at early postnatal stages and exhibit hematopoietic defects and cerebellar hypoplasia. Not surprisingly, the MDM2/p53 axis plays a crucial role in several neuronal processes, by modulating neuronal survival, cell cycle (including apoptosis and senescence), and stress responses [12]. Nevertheless, to the best of our knowledge, the deep interaction and functionality of this axis has not been evaluated in AS neuronal models yet.

Under normal circumstances, autophagy and UPS processes collaborate to remove abnormal proteins, but any dysfunction in one of this processes can lead to defects in neuronal development and neuroinflammatory processes [13,14]. In this sense, the accumulation of misfolded proteins has been widely studied in neuronal diseases: proteins that fail to fold properly—likely due to an imbalance in proteostasis—can accumulate both intra- and extracellularly, leading to toxicity, neuroinflammation, and neuronal cell death. This phenomenon primarily affects the brain and is a hallmark of several neurological disorders [15,16,17,18]. Interestingly, overexpression of UBE3A promotes degradation of mutant Huntingtin [19] via UPS. UBE3A was also reported to promote the proteasomal degradation of misfolded polyglutamine and Hsp70-bound proteins [20,21]. However, so far, there is no investigation on the misfolded proteins in AS in the literature.

On this basis, the objective of our study was to dissect the involvement of the ubiquitin–proteasome/autophagy system in the regulation of intracellular signaling pathways related to misfolded protein accumulation in AS neuronal cells. UBE3A-silenced SH-SY5Y neuroblastoma cells represent an optimal in vitro cellular model for AS, already used and demonstrated [22,23]. Here, AS neuronal SH-SY5Y cell and murine models (UBE3A^m−/p+^) [24] were employed to dissect the autophagy process and the related signaling pathways (i.e., AKT-mTOR, p53), the functionality of the MDM2–p53 axis, and the accumulation of misfolded proteins.

## 2. Results

### 2.1. Involvement of the Autophagy System in the AS Model

**Figure 1:**
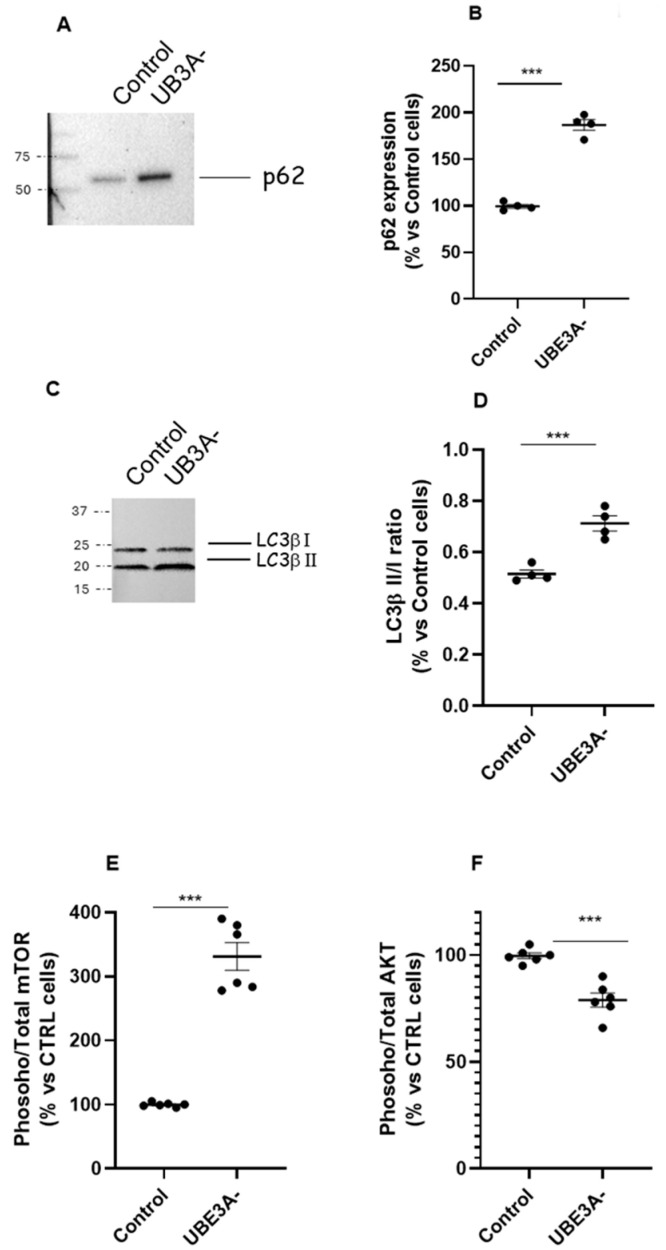
Autophagy and mTOR/AKT pathway in AS neuronal cells’ model. (**A**–**D**) Cell lysates from control and UBE3A^−^ SH-SY5Y cells were used for western blotting analysis using specific antibodies to monitor autophagy activation, i.e., p62 (panel **A**) and LC3b (I and II, panel **C**). A ‘stain-free protein normalization’ method was used for the normalization of bands to total protein in blots, eliminating the need for housekeeping proteins. The data are expressed as % vs. control cells (for p62) and as ratio LC3b II/LC3b I, and represent the mean ± SEM of four independent experiments. Comparisons between control and UB3A^−^ were performed using an unpaired two-tailed Student’s *t*-test; *** *p* < 0.001. (**E**,**F**) SH-SY5Y (control and UBE3A^−^ cells) were fixed, and specific antibodies (total mTOR and phospho-mTOR, (panel **E**); total AKT or phospho-AKT, (panel **F**)) were used in specific immunoezymatic assays, as reported in Section 4. The data are expressed as the ratio between phosphorylated and total protein levels (in % vs. control cells) and represent the mean ± SEM of at four independent experiments. Comparisons between control and UBE3A^−^ were performed using an unpaired two-tailed Student’s *t*-test; *** *p* < 0.001 vs. control cells.

We investigated the autophagy process in wild-type (WT) and UBE3A-silenced (AS model; from now on UBE3A^−^) SH-SY5Y neuroblastoma cells. A western blot analysis was performed in SH-SY5Y cells to verify the expression of key proteins involved in autophagy (i.e., LC3β and p62) under UBE3A silencing. Both p62 (Figure 1A,B) and the ratio LC3β II/LC3β I (Figure 1C,D) were significantly enhanced in UBE3A^−^ cells in respect to control cells, thus indicating the induction of autophagic process.

Next, the expression of the phosphoinositide 3-kinase (PI3K)/protein kinase B (AKT)/mammalian target of rapamycin (mTOR) pathway was examined by immunoassays, as critical regulators of autophagy. The level of active mTOR (activated mTOR/total mTOR) resulted in significantly augmented UBE3A^−^ cells (Figure 1E). In contrast, the ratio between activated AKT/total AKT was significantly diminished in AS cells with respect to control cells (Figure 1F). Similar results were obtained by determining the AKT/mTOR pathway in the cortex of mouse UBE3A^−^, when compared to WT animals (Supplementary Figure S1A,B). Overall, these data suggest the presence of a dysregulation of the AKT/mTOR pathway and in the autophagy process in AS neuronal cells.

### 2.2. Involvement of the UPS in the AS Model

**Figure 2:**
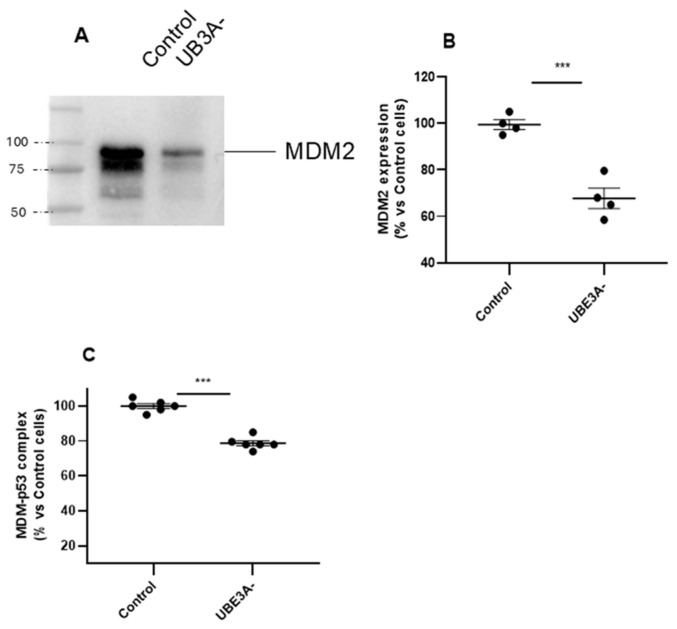
MDM2–p53 axis in the AS neuronal cell model. (**A**,**B**) Cell lysates from control and UBE3A^−^ SH-SY5Y cells were used for western blotting analysis using specific antibodies to MDM2. A ‘stain-free protein normalization’ method was used for the normalization of bands to total protein in blots, eliminating the need for housekeeping proteins. (**C**) Cell lysates from control and UBE3A^−^ SH-SY5Y cells were used to detect MDM2-p53 complex by a specific immunoezymatic assay, as reported in the Section 4. Data are expressed as percentage versus control cells and are mean ± SEM from four independent experiments. Comparisons between control and UBE3A^−^ were performed using an unpaired two-tailed Student’s *t*-test; *** *p* < 0.001 control vs. UBE3A^−^.

Subsequently, UPS was investigated by assessing expression and functionality of the MDM2–p53 axis. The results showed that the ubiquitin ligase MDM2 was downregulated in UBE3A^−^ SH-SY5Y cells (Figure 2A,B). We next investigated the contribution of the tumor suppressor p53, which is normally inhibited by MDM2 by a direct interaction [25]. As depicted in Figure 2C, the amount of MDM2 linked to p53 protein was also significantly diminished in UBE3A^−^ cells. Similar results were obtained by measuring the MDM2–p53 complex in the cortex of AS mice (Supplementary Figure S1C).

**Figure 3:**
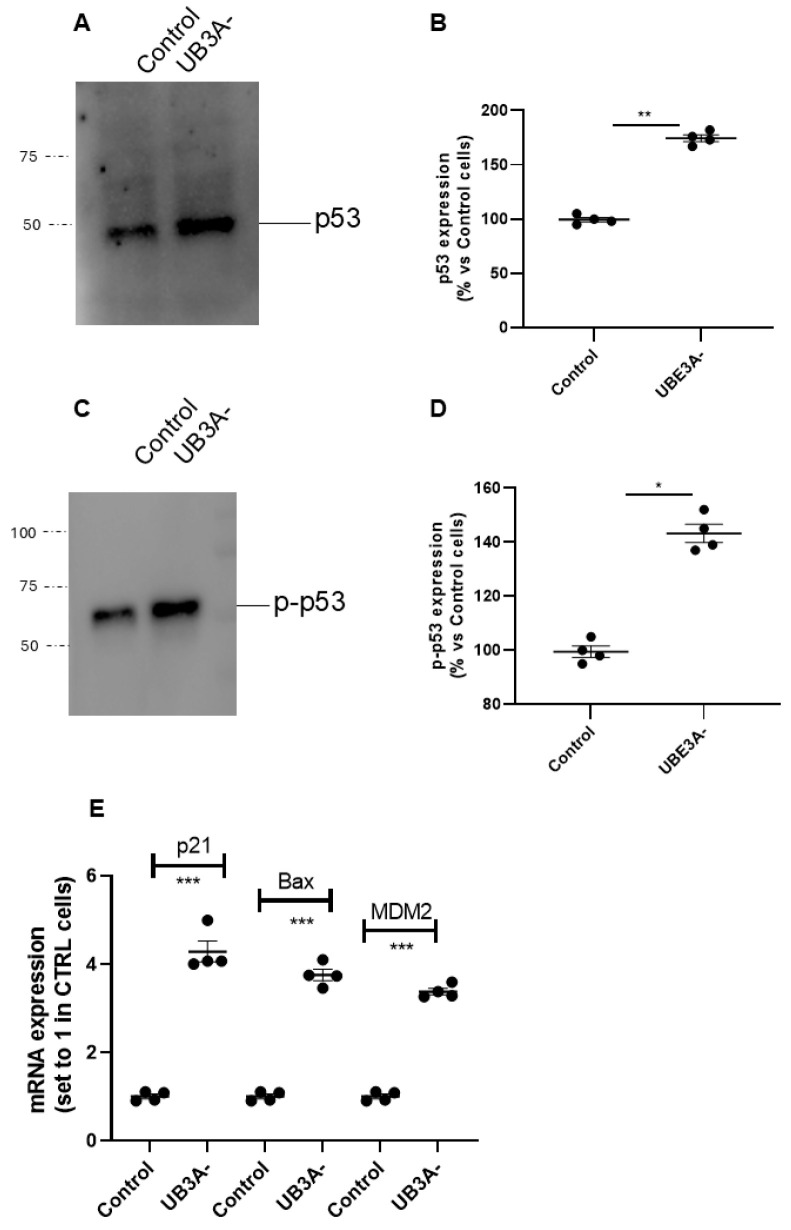
p53 expression and activity in SH-SY5Y AS cellular model. (**A**–**D**) Cell lysates from SH-SY5Y (control and UBE3A^−^ cells), were used for western blotting analysis using specific antibodies to total p53 (**A**,**B**) and phosphorylated p53 (**C**,**D**). A ‘stain-free protein normalization’ method was used for the normalization of bands to total protein in blots, eliminating the need for housekeeping proteins. Data are expressed as percentage versus control cells and are mean ± SEM of at least three independent experiments Comparisons between control and UB3A^−^ were performed using an unpaired two-tailed Student’s *t*-test; * *p* < 0.05, ** *p* < 0.01, UBE3A^−^ vs. control. (**E**) SHSY-5Y (control and UBE3A^−^) cells were collected for RNA extraction and retro transcription to cDNA. A real-time PCR analysis was performed to monitor mRNA expression of p53-traget genes (i.e., Bax, p21, and MDM2). Data are expressed as fold change relative to Control cells (set to 1) and represent mean ± SEM of four independent experiments. Comparisons between Control and UBE3A^−^ were performed using an unpaired two-tailed Student’s *t*-test; *** *p* < 0.001.

Moreover, the expression of the total levels of p53 was increased in UBE3A^−^ cells (Figure 3A,B), and accordingly, the level of phosphorylated p53 (i.e, the active form of the oncoprotein) was significantly augmented in UBE3A^−^ cells (Figure 3C,D).

Consistent with these data, we found that the transcriptional activity of p53 was augmented in UBE3A^−^ cells; in particular, the mRNA levels of p53-target genes (i.e., p21, MDM2, and Bax) were significantly enhanced in UBE3A^−^ cells (Figure 3E) with respect to those in control cells. Overall, we found an increase in the total levels and in the activity of p53 and a decrease in the quote of p53 bound to its negative regulator MDM2 in AS neuronal cells. These data suggest the presence of an enhancement in p53 expression and activation in UBE3A^−^ neuronal cells.

### 2.3. Misfolded Protein Accumulation in the AS Models

Dysfunctions in autophagy and UPS systems can lead to the accumulation of proteins (misfolded and non), mainly related to neuroinflammatory processes.

**Figure 4:**
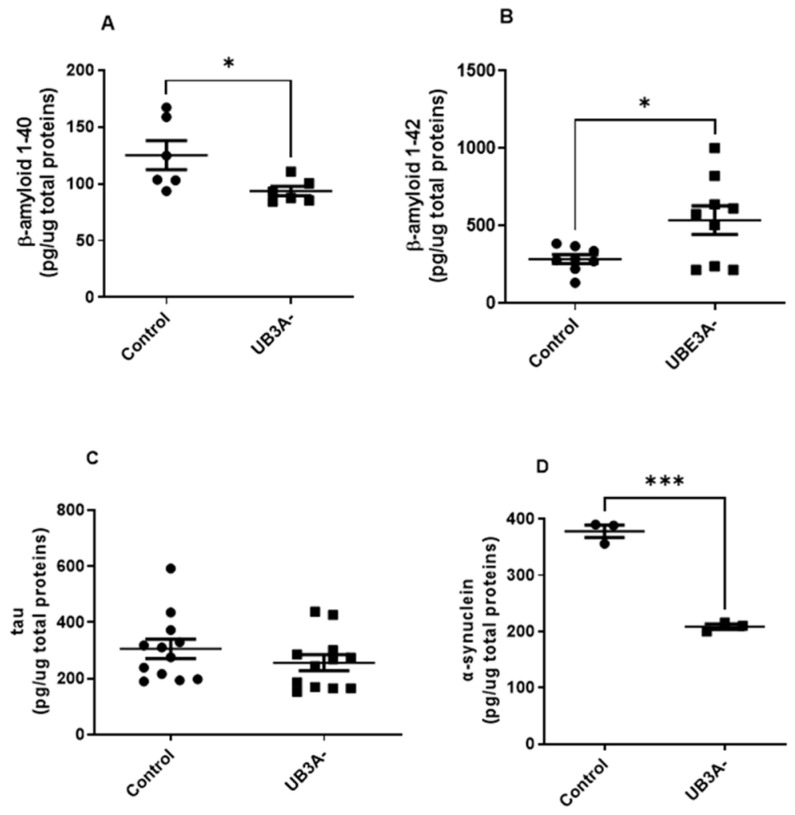
Misfolded proteins levels in the AS cellular model. Cell lysates obtained from SH-SY5Y (control and UB3A^−^) cells were used to detect b-amyloid 1-40 (**A**), b-amyloid 1-42 (**B**), tau (**C**), and a-synuclein (**D**) by specific immunoenzymatic assays, as reported in Section 4. The data are expressed as pg/µg total proteins (mean ± SEM of N = 4 independent experiments performed in duplicate). Comparisons between control and UBE3A^−^ were performed using an unpaired two-tailed Student’s *t*-test; * *p* < 0.05, *** *p* < 0.001.

In our hands, cells deficient for UBE3A presented significantly lower levels of the shorter form of Amyloid-β (Aβ) protein (i.e., Aβ_1-40_) and higher levels of Aβ_1-42_, with respect to control cells (Figure 4A,B). Similar results were also evidenced in cortexes of adult AS mice with respect to their WT littermates (Supplementary Figure S2A,B). In contrast, oligomeric α-synuclein (α-syn) levels were significantly reduced in both UBE3A^−^ SH-SY5Y cells and murine AS cortex lysates (Figure 4C and Supplementary Figure S2C). Finally, similar tau levels were evidenced in control and UBE3A^−^ samples, both in SH-SY5Y cells (Figure 4D) and the murine brain cortex (Supplementary Figure S2D). Overall, these data indicate the presence of different levels and processing of misfolded proteins in UBE3A^−^ neuronal cells.

### 2.4. Morphological Changes in the AS Cellular Model

**Figure 5:**
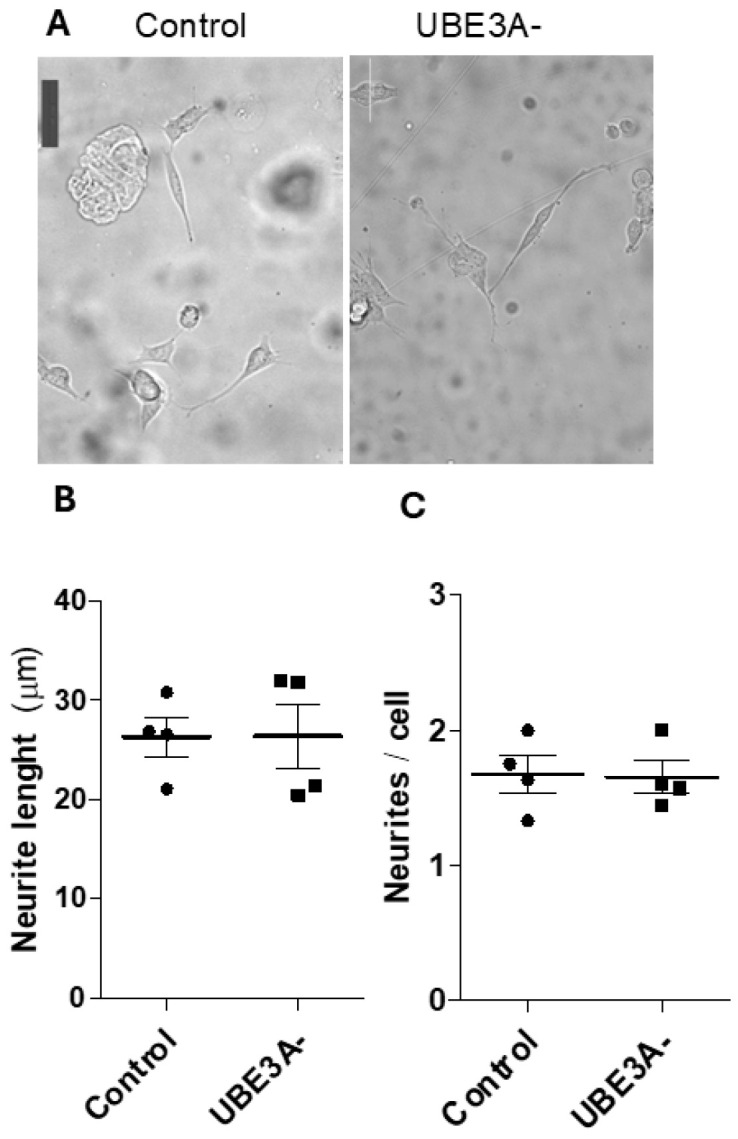
Morphological changes of AS cellular model. (**A**) Bright-field images of control and UBE3A^−^ SH-SY5Y cells cultured for 24 h on standard culture plates; scale bars = 50 μm. The mean neurite length (µm) (panel **B**) and number of neurites/cell (panel **C**) were measured in control and UBE3A^−^ SHSY5Y cells, as reported in the Methods section (mean ± SEM of N = 3 independent experiments performed in duplicate).

Ultimately, we verified if UB3A deletion can produce effects on neuronal morphology. The results demonstrate no significant effects on neuronal development (i.e., neurite length, neurite number/cell) when UBE3A was lost in SHSY5Y cells (Figure 5). These data suggest that AS neuronal cells do not show evident abnormalities at the morphological level in vitro.

## 3. Discussion

In the present study, we showed that the loss of UBE3A in neuronal cells is associated with alterations in the autophagy process and in the ubiquitin–proteasome system, leading to the accumulation of proteins related to neuroinflammation and neuronal impairment. In particular, the main findings are as follows: (i) autophagy markers (p62 and LC3β) are upregulated in UBE3A^−^ cells in respect to control cells; (ii) a dysregulation of the AKT/mTOR pathway is present in both AS neuronal cell and murine cortex samples; (iii) the tumor suppressor protein p53 is significantly enhanced in terms of expression and transcriptional activity in UBE3A^−^ cells, and in parallel the ubiquitin ligase MDM2, which normally degrades it by a direct interaction, is downregulated in UBE3A^−^ cells; (iv) AS neuronal cells present a significant alteration in the ratio levels of Amyloid-β (Aβ_1-40_/Aβ_1-42_) with respect to control cells, as well as a significant reduction in α-syn levels, thus evidencing an accumulation of toxic proteins related to neuroinflammation processes. Nevertheless, we confirmed that AS neuronal models do not show evident abnormalities in vitro, in particular at the morphological level. Collectively, these data lead us to propose that UBE3A loss in AS neuronal cells induces an alteration in the autophagy and UPS system signaling pathways, thus leading to the accumulation of toxic proteins that in turn could impact neuronal functioning and also synaptic development.

UBE3A is a critical player of UPS, which is involved in neuronal morphological maturation, development, and synaptic plasticity [25,26,27], and also in the regulation of autophagy. We found LC3β and p62 upregulated in UBE3A^−^ cells. Consistently, the literature has evidenced that UBE3A deficiency is associated with dysregulated autophagy in cerebellar Purkinje cells [7] and in the hippocampus of AS mice [28]. In general, in AS, a deregulated, likely increased autophagy is reported [7], in agreement with our results.

In our hands, an enhancement in mTOR activation and a significant diminishment in the activation of AKT were evidenced in both AS neuronal cells and mouse cortexes, thus suggesting a dysregulation of the AKT/mTOR pathway, which is in turn a critical regulator of autophagy [29]. Similarly, other studies have revealed that mTOR signaling is abnormal in the hippocampus [30] and in the cerebellum of the AS mouse model [31]; in particular, the authors have shown an intriguing imbalance in the activation of mTOR complexes, with mTORC1 overactivated and mTORC2 underactivated (with the former controlling protein synthesis and cell proliferation and the latter regulating cytoskeleton remodeling and cell survival) [32]. Consistently, mTORC1 inhibition or mTORC2 activation, with the consequent reactivation of AKT signaling, has been shown to improve hippocampal synaptic plasticity and learning in AS mice [30]. Our results show an increase in mTOR phosphorylation and reduced AKT activation, as in [31]. The increased activated mTOR levels in our results could be involved, at different levels, in mTORC1 or in mTORC2 signaling, thus leading to different effects on autophagy and cross-talking in different ways with AKT, and in line with the reported imbalance between mTORC1 and mTORC2 activity. The involvement of other pathways, upstream of mTORC1-2 or AKT, cannot be excluded. The reduced AKT activation is in agreement with the presence of a dysregulation, likely overactivation, of autophagy [31]. To further increase the complexity of this intracellular landscape, AKT also activates by phosphorylation of MDM2; therefore, the reduced levels of active AKT can also explain the reduced activity of MDM2 on p53 [33]. Moreover, p53 activity itself can exert a negative feedback loop on AKT activation [34].

Both UBE3A and MDM2 have been shown to function as an E3 ligase in the UPS, for example targeting p53, regulating proteostasis in physiological and pathological conditions [25]. Herein, MDM2 was downregulated in UBE3A^−^ SH-SY5Y cells, and we also found a significant decrement in the quote of MDM2 interacting with p53 level [25]. Consistently, the expression of total and activated p53 was enhanced in AS neuronal cells. Our results demonstrate that UBE3A deficiency is associated with the increase of total p53, as already reported [24], and with the decrease in the quote of p53 bound to its negative regulator MDM2. Moreover, the transcriptional activity of p53 was augmented in UBE3A-silenced neuronal cells, overall confirming an enhancement of p53 expression and activation in UBE3A^−^ cells. Because the MDM2 gene is a direct target of p53, MDM2 transcription was enhanced in UBE3A^−^ cells as part of a negative feedback mechanism [35]. In supporting our findings, the literature has reported that UBE3A-deficiency-induced autophagy is associated with activation of the p53 pathway [9]. Generally, p53 can be involved in the impairment of neuronal differentiation/survival and synaptic development that is evidenced in AS [36]. In this sense, the activation of p53-dependent apoptosis has been demonstrated to impede the growth of the embryonic cerebral cortex in an autism spectrum disorder mouse model [37].

Dysregulations in autophagy and UPS processes in proteostasis can lead to defects of neuronal development and neuroinflammatory processes [13,14]. In our hands, AS SH-SY5Y cells and the murine cortex presented significantly higher levels of Aβ_1-42_, the most toxic and aggregate Aβ form, with respect to the respective control samples; in parallel, AS samples showed lower levels of the shorter Aβ_1-40_ form. Interestingly, autism spectrum disorders (ASDs) are associated with enhanced processing of amyloid-β precursor protein (APP) by secretase-α, and intraneuronal accumulation of N-terminally truncated Aβ peptides in the brain cortex [38], thus confirming the involvement and the accumulation of amyloidogenic peptides also in neurodevelopmental disorders. Recently, Frackowiak and Mazur-Kolecka have suggested that ASD, epilepsy, and self-injurious behaviors all contribute to the enhanced production and accumulation of Aβ peptides, which in turn cause and further enhance dysfunctions of the neuronal networks (i.e., that manifest as autism clinical symptoms, epilepsy, and self-injurious behaviors) [38].

In contrast, oligomeric α-synuclein (α-syn) levels were significantly reduced in both AS SH-SY5Y cells and AS mouse cortexes. These data are in accordance with previous studies conducted on ASD subjects. The literature has recently suggested synucleins as biomarkers of severity in ASD, in particular, the fold decrease in levels of α-synuclein as discriminative for the diagnosis and severity with good sensitivity and specificity [39]. Finally, comparable levels of tau, am MAP involved in cytoskeleton regulation, were evidenced in UBE3A-silenced models with respect to controls.

Ultimately, we looked at possible effects on neuronal morphology. To the best of our knowledge, AS neuronal models do not show evident abnormalities in vitro, in particular at the morphological level. SH-S5SY neuronal cells have normal development, neurite number, or length. UBE3A-deficient SH-SY5Ys showed morphological differences in vitro only if cultured on micro-structured substrates with directional topographical cues (i.e., micro-grooved substrates); there, AS neuronal cells showed impaired neurites’ alignment to the micro-tracks and impaired focal adhesion, morphological development, and intracellular signaling [23]. However, the presence of abnormalities in AS neurons is emerging at several levels in vivo (e.g., neuronal axonal initial segment, brain size, excitatory–inhibitory balance), even if with some conflicting outcomes about the presence of abnormalities at the structural level (e.g., synaptic density, dendritic arborization) [24,40,41,42].

Overall, UBE3A-deficient neuronal cells exhibit deficits in several intracellular pathways involved in or interacting with proteostasis and protein degradation. These results are in line with the view of UBE3A as a ‘molecular tightrope’ in neurons [43], as suggested by the several mechanisms in place to continually adjust neuronal UBE3A activity (genetic imprinting, stimulation by cancer-causing E6 virus [44], phosphorylation via PKA [45]) and the range of different neurodevelopmental disorders due to aberrant levels or functioning of UBE3A (AS, autism, epilepsy, intellectual disabilities).

## 4. Materials and Methods

### 4.1. Neuronal Cell Cultures

For in vitro model studies, we employed the SH-SY5Y human neuroblastoma-derived cell line. We used wild-type (WT) and UBE3A-silenced (AS model, UBE3A^−^) SH-SY5Y cells; both cell lines were generously provided by Prof. M. Scheffner [22,23]. Cells were cultured in DMEM/F-12 medium supplemented with 10% fetal bovine serum (FBS) and 1% penicillin/streptomycin. For the UBE3A^−^ cells, 1 µg/mL puromycin (P7130, MERCK, Darmstadt, Germany) was added during each medium change to maintain selective pressure. Culture medium was refreshed three times per week, and cells were passed upon reaching approximately 80% confluence, following previously established protocols [23,46].

### 4.2. Mouse Model of AS

Mice used in this study were C57BL/6 mice with Ube3a maternal deletion (B6.129S7-Ube3a tm1Alb/J; Stock No. 016590, The Jackson Laboratories [47]). Ube3am+/p− females were crossed with wild-type males (all mice in 129S background), and pups, which were either wild-type (WT) or Ube3a m−/p+ (designated as AS), were used for experiments; genotyping was performed as in [46]. Animals were housed in standard cages in a room maintained at 22 + 1 °C with a 12 h light–dark cycle. They had ad libitum access to food (standard diet, 4RF25 GLP Certificate, Mucedola, Settimo Milanese, Italy) and water. All authorization documents were approved by The Italian Ministry of Health (Authorization n. 582/2021-PR del 23/07/2021), in compliance with the directive of the European Communities Council 63/2010. Both male and female mice (n = 12 in total, with 6 WT control mice and 6 AS mice, from four different litters) were sacrificed (at P110 ± 5 days) by cervical dislocation; the brains were immediately extracted, sectioned, and frozen in dry ice before storage at −80 °C.

### 4.3. Sample Lysate Preparation for Immunoenzymatic Assays

SH-SY5Y cells at subconfluence were washed in ice-cold phosphate-buffered saline (PBS 1x), scrapped, and collected by centrifugation, and resuspended in Lysis buffer (20 mM Tris HCl, 137 mM NaCl, 10% glycerol, 1% NONIDET40, 2 mM EDTA, pH 8), containing 1% of the protease inhibitor cocktail (Sigma Aldrich, Milan, Italy). For cortex tissues, the samples were cut into approximately 30 mg of tissue, homogenized in the same lysis buffer as above, and sonicated on ice for 30 s, three times. The lysates were then centrifuged at 15,000× *g* for 15 min at 4 °C to eliminate residues. The supernatant was collected, and protein concentration was determined to ensure loading of 30 µg of protein per well.

Optimal composition of lysis buffer, as well as reaction conditions, was determined in preliminary experiments [48].

### 4.4. Protein Expression by Western Blot Analysis

Mouse cortexes were cut into ~30 µg of tissue followed by the lysis in RIPA buffer (9.1 mM NaH2PO4, 1.7 mM Na2HPO4, 150 mM NaCl, pH 7.4, 0.5% sodium deoxycholate, 1% Nonidet P-40, 0.1% SDS, and a protease-inhibitor cocktail) with sonication (amplitude 50%, 3 times for 30 s each), then the samples were lysed for 2 h at 4 °C.

For SH-SY5Y cells, the cell pellet was lysed following the same protocol as for brain tissues, except for the sonication amplitude that was used at 35%. After the protein quantification, equal amounts of protein (~40 µg of protein) were diluted in Laemmli solution and loaded in a precast gel of SDS-PAGE (7.5%), then transferred to PVDF membranes (Bio-Rad, Milan, Italy). The membrane was incubated in a blocking solution of 5% milk in TBS-0.1% Tween for 60 min, and then the primary antibodies were incubated overnight at 4 °C, diluted in a 5% milk TBS-Tween solution. Then, the next day, membranes were washed out in TBS-0.1% Tween solution 3 times for 5 min each, followed by incubation in the corresponding secondary antibody for 2 h at a room temperature (at a shaker).

The primary antibody detection and the acquisition of the blot images were performed using a chemiluminescent substrate (ECL, Bio-Rad). The following primary antibodies were employed: anti-MDM2 (sc-5304, Santa Cruz Biotechnology, Santa Cruz, CA, USA, diluted 1:500); p-62 (SQSTM1/p62, #5114, Cell Signaling Technology, Danvers, MA, USA, diluted 1:1000); LC3β (sc-271625, Santa Cruz Biotechnology, Santa Cruz, CA, USA, diluted 1:500); total p53 (sc-126, Santa Cruz Biotechnology, Santa Cruz, CA, USA, diluted 1:500); Phosphorylated p-p53 (sc-101762, Santa Cruz Biotechnology, Santa Cruz, CA, USA, diluted 1:500).

The densitometric analysis of the immunoreactive detected bands was performed using Image Lab Software (Version 6.1, Bio-Rad, Milan, Italy). The data were reported as the percentage compared to the control (WT), set as 100%. All the western blot analyses were performed using the ChemiDoc XRS+Gel Imaging System (Biorad, Milan, Italy), which comprises the use of stain-free total protein normalization. This method of normalization takes into account the intensity of all proteins in the lane, the sample loading variations, variations during electrophoresis, and also variations during transfer, thus eliminating the use of housekeeping proteins (1), as previously reported [49]. Densitometric analysis of immunoreactive bands was elaborated by ImageJ (Version 1.54p, NHI, USA). Data are reported in % vs. control samples.

### 4.5. MDM2–p53 Complex in AS Models

To analyze the formation of the MDM2–p53 complex, we performed an immunoenzymatic assay, as previously described [48], using approximately 30 µg of mouse cortex tissue or SH-SY5Y cell lysate samples (Sigma-Aldrich, Merck, St Louis, MO, USA). Briefly, plates were coated overnight at 4 °C with a rabbit monoclonal antibody for MDM2 (#515415, Cell Signaling, Danvers, MA, USA), diluted 1:50 in 0.1 mg/mL polyornithine. Following a blocking step for non-specific sites performed for 2 h at 37 °C using 1% Bovine Serum Albumin (BSA), samples were added to the plate and incubated for 1 h at room temperature. Primary and secondary antibodies, diluted in a solution of 5% of milk in TBS-Tween, were applied and incubated for 1 to 1.5 h at 37 °C. For the primary antibody, we used a mouse monoclonal antibody for p-53 (#sc-126, Santa Cruz, CA, USA; diluted 1:500), and for the secondary antibody we used an anti-mouse HRP-conjugated antibody (diluted 1:3000). Between each step, plates were washed with PBS containing 0.01% Tween. After the 3,3′,5,5′-Tetramethylbenzidine (TMB) step and color development, the absorbance was read at 450 nm. The results were expressed as percentages relative to controls (WT SH-SY5Y cells or WT mice).

### 4.6. Total and Phosphorylated AKT/mTOR in AS Models

For the study of the AKT/mTOR signaling pathway, an in-house immunoenzymatic assay was performed using lysates obtained from SH-SY5Y cells and mice cortexes. Cells were washed in PBS 1x and fixed with 2% formaldehyde and incubated at room temperature (RT) for 20 min. As concerns brain homogenates, 30–50 mg of tissue were lysed in Lysis buffer (see above) and centrifuged to eliminate residue. Equal amounts of tissue lysates (30–40 µg) were added in a 50 µL/well of Na_2_CO_3_ buffer for 2 h at 37 °C, pH 9.6. A quantity of 100 µL di PFA 4% in PBS was added as fixing solution for 30 min at room temperature. After washing, experiments were conducted for cell lysates as follows.

After, a quenching buffer solution (10% of hydrogen peroxide, 100x Sodium Azide in PBS 1X) was added and incubated at RT for 20 min. Next, a blocking step was performed for 1 h (1% BSA and 0.1% Triton X-100 in PBS 1x) at RT in a shaker. Between each step, the plate was washed with a wash buffer solution (10% Triton X-100 in PBS 1x) for 5 min, three times. The primary antibodies (total AKT, Antibody, sc81434; phospho-AKT (Ser473), sc7985, both Santa Cruz Biotechnology, Santa Cruz, CA, USA; mTOR Antibody #2972; Phospho-mTOR (Ser2448) Antibody #2971, both Cell Signaling Technology, USA) were diluted at 1:300 in a 1% BSA-0.03% Triton X-100 solution at 4 °C overnight. After the incubation for 2 h of the secondary antibody, a last wash step was performed twice with wash buffer solution for 5 min and twice with PBS 1x for 5 min for each one. Following TMB addition and color development, the absorbance was read at 450 nm. The data were expressed as percentages relative to control SH-SY5Y cells or control WT mice, each set to 100%.

### 4.7. RNA Extraction and Real-Time PCR Analysis

SH-SY5Y cells (control and UB3A^−^) were collected, and total RNA was extracted using a RNeasyH Mini Kit (Qiagen, Hilden, Germany) according to the manufacturer’s instructions. cDNA synthesis was performed with 500 ng of RNA using the i-Script cDNA synthesis kit (BioRad, Hercules, CA, USA). RT-PCR reactions consisted of 25 µL of FluocycleH II SYBRH (Biorad, Milan, Italy), 1.5 µL of both 10 mM forward and reverse primers, 3 µL of cDNA, and 19 µL of H_2_O [50]. The reactions were performed for 35 cycles. Sequences and annealing temperatures have been reported before [51].

### 4.8. Misfolded Protein Accumulation in Angelman Syndrome Models

To detect misfolded proteins in SH-SY5Y cells and mouse cortexes, an in-house indirect quantitative immunoenzymatic assay was performed [52,53,54,55]. Details regarding the antibodies and their respective dilutions are provided in Table 1.

Plates were coated overnight at 4 °C with antibodies diluted in 0.1 mg/mL polyornithine. Following a blocking step for non-specific sites performed for 2 h at 37 °C using 1% Bovine Serum Albumin (BSA), samples and standard curve dilutions were added and incubated for 2 h at room temperature. Primary and secondary antibodies, diluted in a solution of 0.2% BSA in PBS with Triton X-100, were applied and incubated for 1 to 1.5 h at 37 °C. Between each step, plates were washed with PBS containing 0.01% Tween. After the TMB step and color development, the absorbance was read at 450 nm. The standard curve was constructed using a serial dilution of a commercial human recombinant protein at eight different concentrations.

### 4.9. Cellular Morphology in the AS Cellular Model

SHY5Y neuronal morphology was quantified by measuring neurites, at 24 h from seeding; a minimum threshold of 10 μm for neurite length was imposed for this analysis. Living-cell imaging was performed using an inverted Nikon-*ti* PSF widefield microscope (Nikon, Tokyo, Japan), with a 20x NA objective and equipped with an incubated chamber coupled to the microscope (Okolab, Pozzuoli, Italy). Morphometric data were collected using ImageJ (National Institute of Health, Bethesda, MD, USA). Neurites (≥10 μm) were semi-automatically segmented (from the point of origin at the perimeter of the cell body to the tip of the neurite growth cone) using NeuronJ, a plugin of ImageJ, and analyzed as previously reported [23]. Data are reported as average value ± SEM, from n ≥ 4 independent experiments for each condition (at least 15 cells/sample were analyzed).

### 4.10. Statistical Analysis

All statistical analyses were performed using GraphPad Prism 10.4.1 (GraphPad Software, San Diego, CA, USA). Data are expressed as mean ± SEM, from at least n = three independent experiments. Differences between control and UBE3A^−^ (AS) samples were assessed using an unpaired two-tailed Student’s *t*-test. A *p* value < 0.05 was considered statistically significant. Statistical significance is indicated as follows: * *p* < 0.05, ** *p* < 0.01, *** *p* < 0.001, **** *p* < 0.0001 vs. control.

## 5. Conclusions

In conclusion, in the present paper we show that AS is characterized by a dysregulation in the autophagic process, which involves a disbalance in the AKT/mTOR axis. Moreover, the MDM2 ligase was downregulated in AS neuronal cells as well, linked to an enhancement of p53 expression and activity. As a result, we demonstrated the presence of an accumulation of toxic amyloidogenic proteins in AS neuronal cells and in cortexes of AS animals. Nevertheless, AS neuronal models do not show evident abnormalities in vitro, in particular at the morphological level.

## Figures and Tables

**Table 1:** Antibodies and dilutions used for misfolded protein detection on Angelman Syndrome models.

Protein	Coating Antibody	Primary Antibody	Secondary Antibody
Aβ_1-42_	#44-344, Invitrogen (Carlsbad, CA, USA) Rabbit polyclonal antibody Dilution 1:1000	#sc-28365, Santa Cruz Biotechnology Inc Mouse monoclonal antibody Dilution 1:200	#31430, Invitrogen Goat anti-mouse IgG (HRP) Dilution 1:5000
Aβ_1-40_	#sc-53828, Santa Cruz Biotechnology mouse monoclonal (aa 1-200 APP695)- for coating Dilution 1:100	# 512700 Invitrogen rabbit monoclonal antibody Dilution 1:1000	#A0545, Sigma-Aldrich Goat anti-rabbit IgG (HRP) Dilution 1:5000
t-tau	#sc-32274, Santa Cruz Biotechnology Mouse monoclonal antibody (recognizing C-terminal) Dilution 1:100	ab109392, abcam (Cambridge, UK) Rabbit polyclonal antibody Dilution 1:1000	#A0545, Sigma-Aldrich Goat anti-rabbit IgG (HRP) Dilution 1:5000
α-syn	NBP2-15365, Alpha-Synuclein Antibody Rabbit polyclonal antibody Dilution 1:100	# sc-12767, Santa Cruz Mouse monoclonal antibody Dilution 1:200	#31430, Invitrogen Goat anti-mouse IgG (HRP) Dilution 1:5000

## Data Availability

The original contributions presented in this study are included in the article/Supplementary Material. Further inquiries can be directed to the corresponding author.

## Supplementary Materials

The supporting information can be downloaded at https://www.mdpi.com/article/10.3390/ijms262211032/s1.

## Abbreviations

The following abbreviations are used in this manuscript:

AS	Angelman Syndrome
*UBE3A*	Ubiquitin-Protein ligase E3A
UPS	Ubiquitin–proteasome system
JNK	c-Jun-N-terminal Kinase
ASD	Autism spectrum disorder
AMPK-mTOR	Mammalian target of rapamycin
MDM2	Murine double minute 2
AKT	Protein kinase B
PI3K	Phosphoinositide 3-kinase
Aβ	Amyloid-β
α-syn	α-synuclein
