# Supplementary material for: Dysfunction of the Autophagy System and MDM2–p53 Axis Leads to the Accumulation of Amyloidogenic Proteins in Angelman Syndrome Models
Source: Int J Mol Sci. 2025 Nov 14;26(22):11032. doi: 10.3390/ijms262211032 (PMC12652799; doi:10.3390/ijms262211032)
Supplement: Supplementary file 1 [file ijms-26-11032-s001.zip › ijms-3934188-supplementary.pdf]

Article

Dysfunction of the autophagy system and MDM2-p53 axis leads to the accumulation of amyloidogenic proteins in Angelman Syndrome models

Jacqueline Fátima Martins de Almeida[1], Martina Contestabile[1], Ilaria Tonazzini[2], Laura Baroncelli3, Chiara De Cesari [3], Claudia Martini[1], Simona Daniele[1]

1Department of Pharmacy, University of Pisa, 56126 Pisa, Italy;

2Istituto Nanoscienze, Consiglio Nazionale delle Ricerche (CNR) @NESTe, Piazza San Silvestro 12, 56127 Pisa, Italy;

3Istituto neuroscienze, CNR, Via Giuseppe Moruzzi 1, 56124, Pisa, Italy

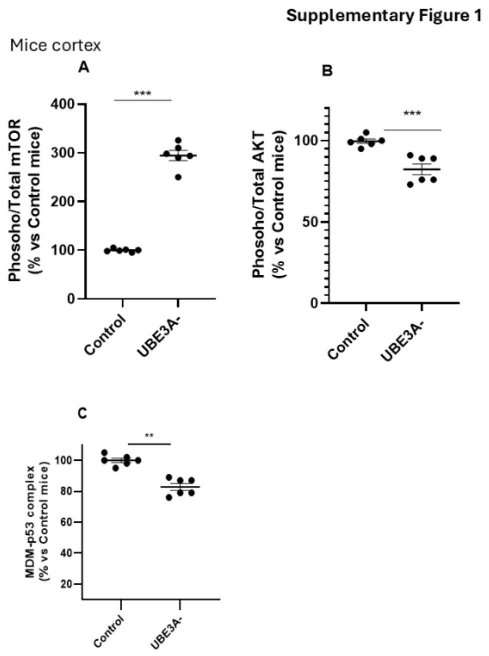

Supplementary Figure S1. mTOR/AKT pathway and MDM2-p53 axis in the AS mice model. (A-B) Cortex lysates from WT and AS mice were tested with specific antibodies (total mTOR and phospho-mTOR, panel A; total AKT or phospho-AKT, panel B) by immunoezymatic assays, as reported in the Methods section. The data are expressed as ratio between phosphorylated/total mTOR or as ratio between phosphorylated/total AKT and reported in % versus the control samples. Data are mean ± SEM of four independent experiments. Comparisons between WT and AS were performed using an unpaired two-tailed Student’s t-test; $^{**}p < 0.01$, $^{***}p < 0.001$. (C) Cortex lysates from WT and AS mice were used to detect MDM2-p53 complex by a specific immunoezymatic assay,

as reported in the Methods section. The data are expressed as percentage versus WT samples and are mean ± SEM of three independent experiments. Comparisons between Control and AS samples were performed using an unpaired two-tailed Student's t-test; **p < 0.01, ***p < 0.001.

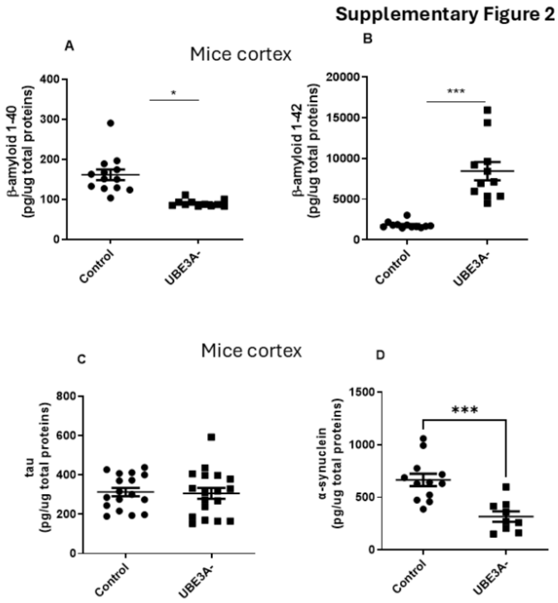

Supplementary Figure S2. Misfolded proteins levels in the AS mice model. Tissue lysates obtained from cortexes of WT and UB3A- animals used to detect β-amyloid1-40 (A), β-amyloid1-42 (B), tau (C) and α-synuclein (D) by specific immunoezymatic assays, as reported in the Methods section. The data are expressed as pg/μg total proteins (mean ± SEM). Comparisons between Control and UB3A⁻ were performed using an unpaired two-tailed Student's t-test. The following symbols were used to indicate significance: *p < 0.05, **p < 0.01, ***p < 0.001, ***p < 0.0001 vs Control.
